# Chest Physiotherapy in Acute Muco-obstructive Lung Disease

**DOI:** 10.7759/cureus.7056

**Published:** 2020-02-20

**Authors:** Ramandeep Kaur, Bhagwan Dass, Abutaleb A Ejaz, Amardeep Singh

**Affiliations:** 1 Medicine, Virginia Commonwealth University, Richmond, USA; 2 Nephrology, University of Florida Health, Gainesville, USA

**Keywords:** mucous plug, chest, physiotherapy, respiratory, pulmonary, dyspnea, x-ray, percussion, therapy, bedside procedures

## Abstract

Acute mucus plugging is a pulmonary emergency associated with increased mortality and often requires rapid bronchoscopic intervention which may not be readily available in all centers. Furthermore, the role and efficacy of alternate conventional measures such as mechanical percussive therapies are uncertain. Herein, we present a patient who rapidly progressed to respiratory distress; a chest X-ray revealed left lobar atelectasis highly suggestive of acute mucus plugging. In the absence of rapid bronchoscopic intervention, bedside chest percussion was initiated with improvement in clinical status. Our case demonstrates the successful utilization of chest physiotherapy in the resolution of acute mucus plugging in an urgent situation and highlights the need for careful attention to respiratory status in high-risk patients who are also undergoing fluid removal with dialysis therapies.

## Introduction

Diseases of the respiratory tract can be associated with unusually thick, inspissated forms of mucus that accumulate within the airways to cause bronchial obstruction. The so-called mucus plugging is not an unusual phenomenon in clinical medicine and the treatment usually requires bronchoscopic intervention [1]. Management of mucus plugs involves proper hydration, bronchodilation, and mucolytic agents. However, little attention is paid to nontraditional risk factors of acute mucus plugging such as dialysis. Fluid removal during dialysis may also be another risk factor for acute mucus plugging in the critically ill. Fluid removal during dialysis may thicken secretions and can precipitate acute mucus plugging. We present a case of a patient on maintenance hemodialysis who developed sudden onset acute mucus plugging and discuss the utility of non-bronchoscopic interventions in emergency situations.

## Case presentation

**Figure 1:**
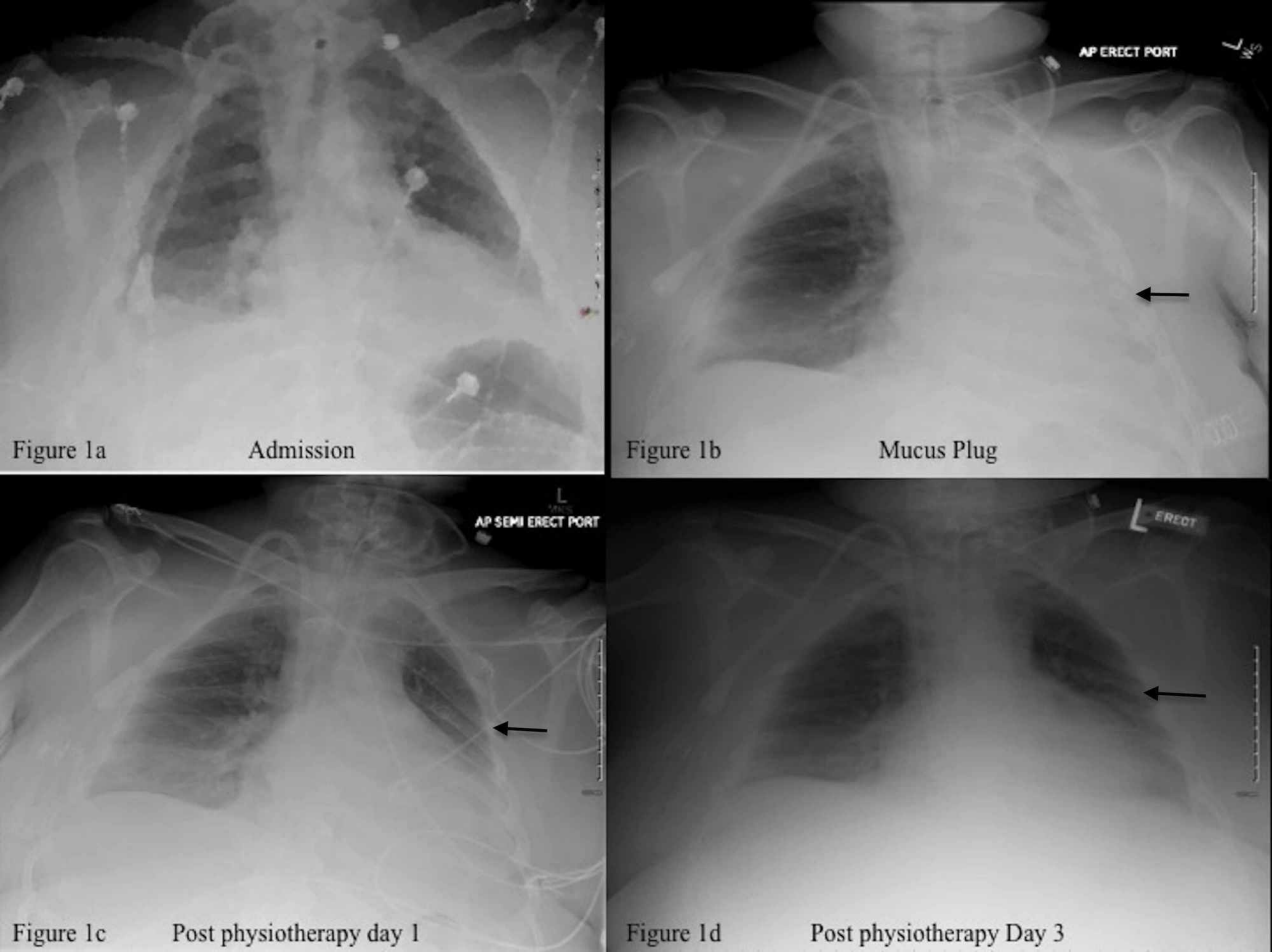
Chest X-rays of the patient's progress before and after intervention 1a) Tracheostomy tube in position of trachea, dialysis catheter in the upper right atrium, cardiomegaly, pulmonary congestion, bilateral pleural effusions, and left lower lobe infiltrates. 1b) Minimal aeration of the left lung, volume loss, and partial atelectasis (arrow). 1c) Improved aeration of left lung post respiratory physiotherapy (arrow). 1d) Normal aeration of the left lung (arrow).

A 42-year-old Caucasian male with non-recovery of recent onset acute kidney injury secondary to sepsis, requiring maintenance dialysis, was transferred from a rehabilitation facility for pulmonary congestion. Physical exam revealed respiration rate of 35 per minute, heart rate 110 beats per minute (bpm), and blood pressure of 110/70 mmHg, patent tracheostomy, bilateral crackles in 2/3 of lung fields on auscultation, and no cardiac rubs or murmurs. Chest X-ray showed pulmonary edema and left lower lobe infiltrates. Due to rapid decompensation of respiratory status, he was intubated via the tracheostomy (Figure 1a). He was treated with antibiotics and underwent several dialysis session in the next several days with significant improvement of his fluid balance status and extubated. However, on the fifth hospital day, he again developed acute respiratory distress. Physical examination now revealed blood pressure 110/70 mmHg, respiratory rate 35/min, heart rate 110 bpm, diminished right lung sounds, and absence of left lung sounds on auscultation. Venous blood gas values at this time were pH 7.21 (7.31-7.41), VpCO2 62.8 (40-52 mmHg), VpO2 42 (30-50 mmHg), and O2 saturation of 66% (>75%). A chest X-ray revealed minimal lung aeration (Figure 1b). An acute bronchial mucus plug was suspected.

The patient was immediately started on mechanical chest percussion therapy while preparations were being made for bronchoscopic interventions. The patient responded to the percussion therapy. Repeat chest X-ray now showed significant partial clearing of the left lung along with a significant increase in aeration of the middle and upper lung (Figure 1c). Bronchoscopy was not required and the patient continued to improve clinically.

A follow-up chest X-ray three days later demonstrated further improvement in lung aeration (Figure 1d). The patient did not have any further clinical complications and was discharged to the outpatient rehabilitation facility.

## Discussion

Mucus is a normally secreted substance of the tracheobronchial tree. In acute respiratory illnesses associated with infection and inflammation, the natural mechanisms of mucus expectoration (i.e., cough) are impaired and lead to accumulation and thickening of the secreted mucus with subsequent bronchial plugging and impaired oxygen exchange [1]. Increased airway microvascular permeability results in bronchial wall edema, swelling and potentially permanent structural changes in airway architecture, and contraction of post-capillary venular endothelial cells with formation worsening of intercellular gaps. Extravasated plasma traverses the epithelium and collects in the bronchial lumen. The loss of epithelial integrity and cilial function reduces mucus clearance and promotes the production of viscous mucus and the formation of luminal mucus plugs [2].

The risk factors for acute bronchial mucus plugging in our patient included excessive mucus production from respiratory tract inflammation, thickening of secreted mucus due to fluid removal with hemodialysis and impaired coughing ability. While maintenance dialysis is essential for volume management for the patient, factors that improve airway clearance, respiratory function and hygiene are important risk factors that need to be optimized to avoid complications. 

The classic chest X-ray finding in mucus plugging is the finger-in-glove sign which most often appears in segmental bronchial atresia and cystic fibrosis but is rare in acquired conditions such as inflammatory or infectious diseases. The common finding in these cases is segmental atelectasis manifesting as hyper-lucency in chest X-ray findings. Computer tomography (CT) is more useful than chest radiography for differentiating between mucoid impaction than other disease processes. The urgency of the patient's clinical condition precluded a CT diagnostic test [3]. 

Bronchoscopic intervention remains the mainstay of treatment of acute mucus plugging, while conventional chest physiotherapy, manually assisted cough, positive expiratory pressure therapy, oscillatory devices, and mechanical insufflation-exsufflation may often bridge the gap from an onset of acute symptoms to definitive bronchoscopic intervention [4]. An evidence-based analysis reported a lack of sufficiently powered randomized, controlled trials to conclusively determine the differences in airway clearance devices versus other airway clearance techniques [5]. Mechanical chest percussions have been successfully used in atelectasis and mucus plugging in spinal cord injury, cystic fibrosis, and asthma patients and are associated with few adverse events [6,7].

## Conclusions

Fluid removal with dialysis is a risk factor for patients at high-risk for acute mucus plugging in the critical care setting. Our case suggests that chest physiotherapy may be a bridge to definitive diagnostic treatment with bronchoscopic intervention in situations when that is not readily available. 

## References

[1] Boucher RC (2019). Muco-obstructive lung diseases. N Engl J Med.

[2] Goldie RG, Pederson KE (1995). Mechanisms of increased airway microvascular permeability: role in airway inflammation and obstruction. Clin Exp Pharmacol Physiol.

[3] Martinez S, Heyneman LE, McAdams HP, Rossi SE, Restrepo CS, Eraso A (2008). Mucoid impactions: finger-in-glove sign and other CT and radiographic features. Radiographics.

[4] McCool FD, Rosen MJ (2006). Nonpharmacologic airway clearance therapies. Chest.

[5] Medical Advisory Secretariat (2009). Airway clearance devices for cystic fibrosis: an evidence-based analysis. Ont Health Technol Assess Ser.

[6] Slonimski M, Aguilera EJ (2001). Atelectasis and mucus plugging in spinal cord injury: case report and therapeutic approaches. J Spinal Cord Med.

[7] Rose L, Adhikari NKJ, Leasa D, Fergusson DA, McKim D (2017). Cough augmentation techniques for extubation or weaning critically ill patients from mechanical ventilation. Cochrane Database Syst Rev.

